# Diverse Staghorn Coral Fauna on the Mesophotic Reefs of North-East Australia

**DOI:** 10.1371/journal.pone.0117933

**Published:** 2015-02-25

**Authors:** Paul Muir, Carden Wallace, Tom C. L. Bridge, Pim Bongaerts

**Affiliations:** 1 Queensland Museum, Townsville, Australia; 2 Queensland Museum, South Brisbane, Australia; 3 ARC Centre of Excellence for Coral Reef Studies, James Cook University, Townsville, Australia; 4 Australian Institute of Marine Science, Townsville, Australia; 5 Global Change Institute, The University of Queensland, St Lucia, Australia; 6 ARC Centre of Excellence for Coral Reef Studies, The University of Queensland, St Lucia, Australia; Leibniz Center for Tropical Marine Ecology, GERMANY

## Abstract

Concern for the future of reef-building corals in conditions of rising sea temperatures combined with recent technological advances has led to a renewed interest in documenting the biodiversity of mesophotic coral ecosystems (MCEs) and their potential to provide lineage continuation for coral taxa. Here, we examine species diversity of staghorn corals (genera *Acropora* and *Isopora*) in the mesophotic zone (below 30 m depth) of the Great Barrier Reef and western Coral Sea. Using specimen-based records we found 38 staghorn species in the mesophotic zone, including three species newly recorded for Australia and five species that only occurred below 30 m. Staghorn corals became scarce at depths below 50 m but were found growing in-situ to 73 m depth. Of the 76 staghorn coral species recorded for shallow waters (depth ≤ 30 m) in north-east Australia, 21% extended to mesophotic depths with a further 22% recorded only rarely to 40 m depth. Extending into the mesophotic zone provided shallow water species no significant advantage in terms of their estimated global range-size relative to species restricted to shallow waters (means 86.2 X 10^6^ km^2^ and 85.7 X 10^6^ km^2^ respectively, p = 0.98). We found four staghorn coral species at mesophotic depths on the Great Barrier Reef that were previously considered rare and endangered on the basis of their limited distribution in central Indonesia and the far western Pacific. Colonies below 40 m depth showed laterally flattened branches, light and fragile skeletal structure and increased spacing between branches and corallites. The morphological changes are discussed in relation to decreased light, water movement and down-welling coarse sediments. Staghorn corals have long been regarded as typical shallow-water genera, but here we demonstrate the significant contribution of this group to the region’s mesophotic fauna and the importance of considering MCEs in reef biodiversity estimates and management.

## Introduction

Reef-building corals host symbiotic microalgae of the genus *Symbiodinium* that provide much of the energy required for their growth and calcification [1]. They are therefore dependent on the high levels of solar radiation normally associated with shallow depths and clear tropical waters [2,3]. However, zooxanthellate corals can extend to depths well in excess of 100 m [4,5], where irradiance levels can be reduced to a fraction (0.07%) of that found at the surface [4]. These deeper coral reef habitats are termed mesophotic coral ecosystems (MCEs), and occur from 30–40 m to the maximum depths of light-dependent coral communities [6]. Due to the legislative limitations associated with SCUBA in this region, relatively little research has been reported from this zone and mesophotic coral diversity remains poorly documented [7, 8]. However, recent improvements in the cost and utility of small remotely operated vehicles (ROVs) and autonomous underwater vehicles (AUVs) have allowed researchers to begin studying depths normally out of range of SCUBA diving. These tools have identified extensive areas of previously undocumented coral reef habitat [9–11]. For example, recent analyses indicate that the area of coral habitat on the Great Barrier Reef (GBR) may be at least 50% larger than currently estimated when MCEs are taken into account [12].

Interest in mesophotic reefs has recently increased, in part due to the prevalence of shallow-water bleaching and the hypothesis that deep reefs may provide a refuge for shallow reef corals during episodic disturbances [7,13,14]. As coral bleaching and storm damage are often shallow reef phenomena e.g. [15, 16], mesophotic populations have the potential to survive and provide a source of larval recruits post-disturbance, potentially enhancing recovery of decimated shallow reef corals. However, the ability of deep reefs to act as a source of recruitment for the shallow reef is largely dependent on the extent of overlap between shallow and deep reef communities. Despite a reasonable understanding of coral community structure over depth in the Caribbean [4,14], little is known about the extent of overlap between shallow reef and mesophotic communities in the Indo-Pacific. Thus, a critical first step in assessing the potential for deep refuge in the region is to determine the species composition of the MCEs.

The staghorn corals (genera *Acropora* and *Isopora*) are one of the main groups of reef-corals, having the largest number of species (122 valid species [17]) and dominating many shallow reef areas around the world [2,18]. Despite their abundance in shallow habitats, reports of staghorn coral species in deeper waters are limited and they are reported to be scarce in mesophotic habitats [4]. Many species in the group also appear particularly susceptible to bleaching, predation by the corallivorous seastar *Acanthaster plancii* and anthropogenic dissolved inorganic nutrients and sediments [19–22]. The IUCN Red List classifies 45% of staghorn species as critically endangered, endangered, threatened or vulnerable with a further 39% data deficient [23], highlighting the need to study this group and their potential refugia. Here, we provide the first account of staghorn coral diversity in the mesophotic zone of the Great Barrier Reef (GBR) and Coral Sea of north-east Australia. For the purpose of this report “mesophotic” refers to depths greater than 30 m and “shallow reef” to depths down to and including 30 m, whereas “depth generalist” refers to species present in both these zones.

## Results and Discussion

**Fig 1:**
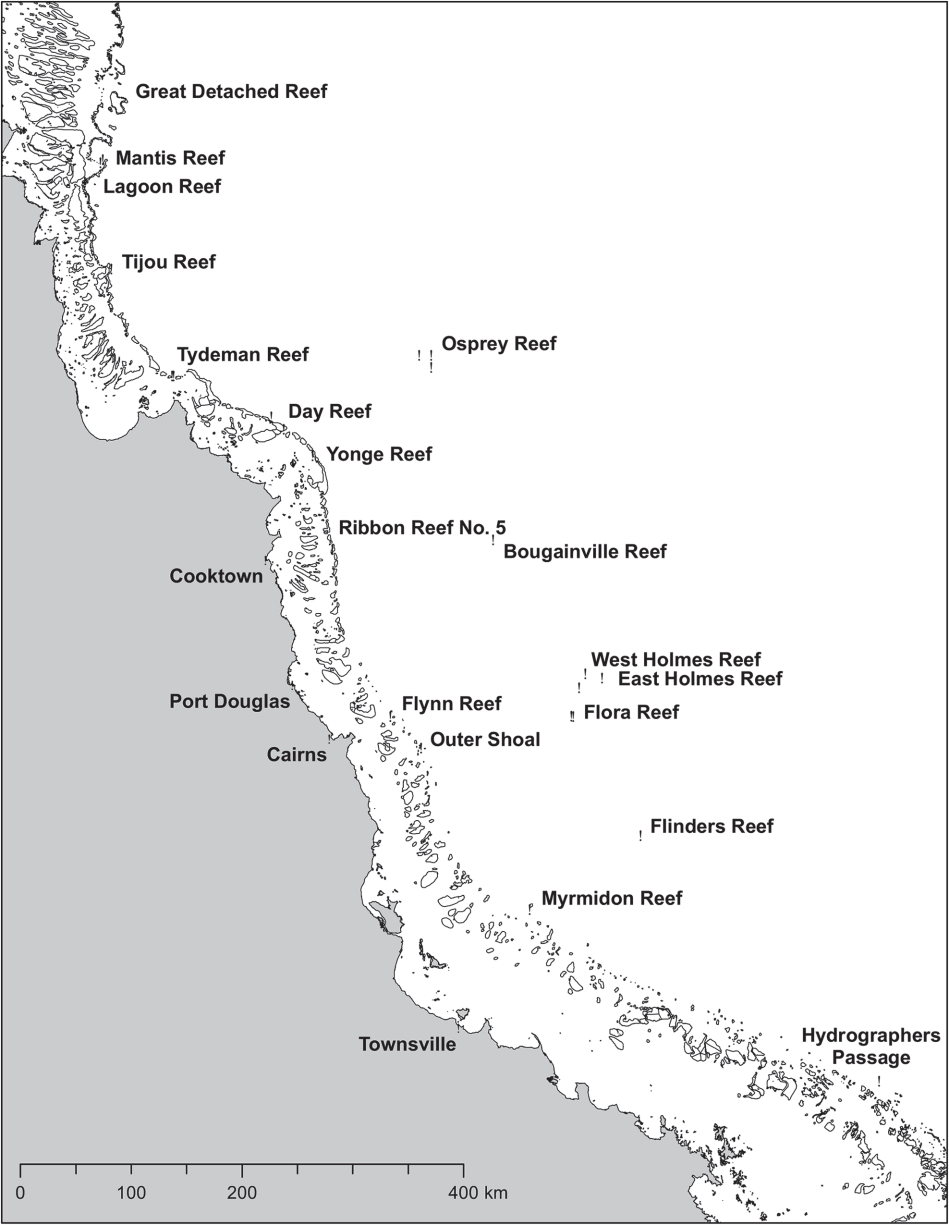
Location of sampling sites.

**Fig 2:**
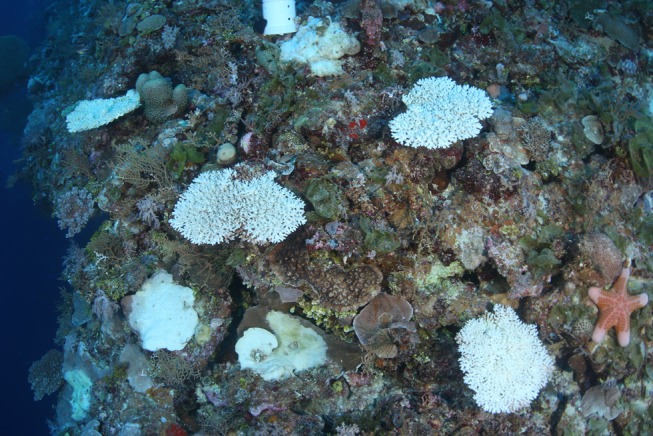
Mesophotic corals at 40 m depth, Flinders Reef, Coral Sea. Staghorn corals make up a significant component of upper mesophotic communities (30–60 m) in the region. Several of the *Acropora* colonies in this photograph are white in colour due to *Acanthaster planci* predation.

**Fig 3:**
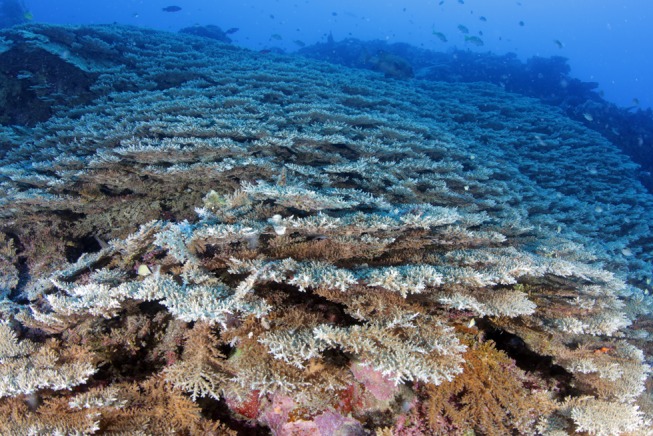
Extensive *Acropora tenella* colony/stand extending from 35 to 45 m depth. This stand at Great Detached Reef, far northern GBR covered over 200 m^2^, comparable to some of the largest monospecific stands of staghorn corals in shallow-reef habitats.

**Fig 4:**
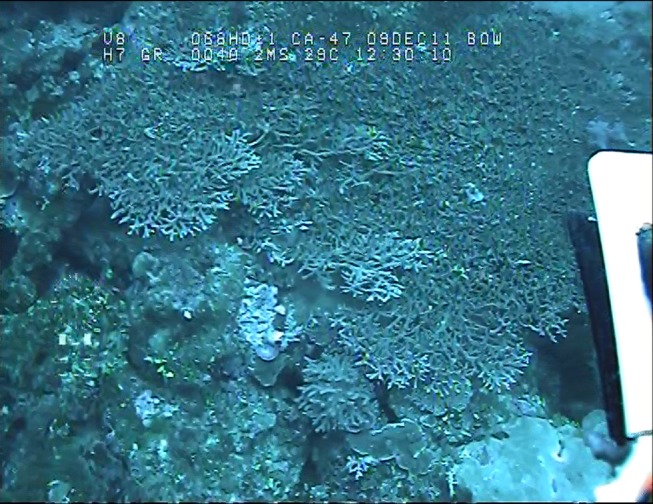
Large *A. donei* colony at 41 m depth, GBR. Photographed and collected at Ribbon Reef No. 5 using an ROV, the collecting grab is visible on the right.

**Fig 5:**
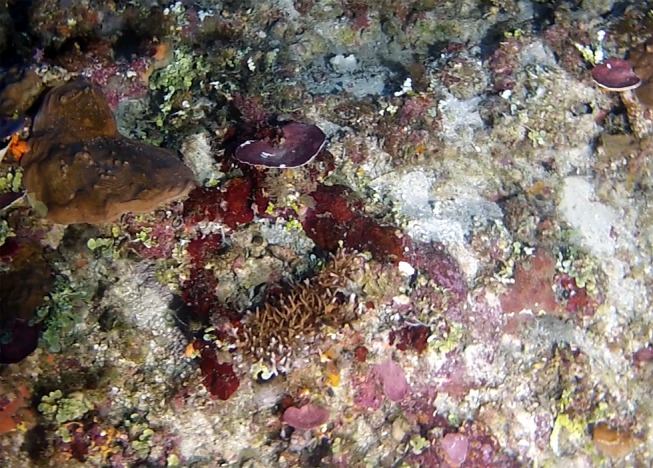
Deepest recorded *Acropora* for the region. *Acropora* colonies became scare and reduced in size beyond 50 m depth but extended to 73 m at some sites. Collected by ROV at 73 m from Day Reef, GBR and designated *cf. A. granulosa* as the sample was small in size and the morphology atypical.

We found a total of 38 staghorn coral species and two potentially undescribed species at mesophotic depths (Table 1, Fig. 1), indicative of a highly diverse staghorn coral fauna in MCEs of the GBR and Coral Sea. Five of these species were deep-water specialists, which were found exclusively below 40 m depth in this study and of these, three are new records for Australia. Prior to this study only four staghorn species have been reported from mesophotic depths of this region (Table 1). In addition to this diversity, we found staghorn corals to be relatively abundant (Fig. 2), capable of forming large mono-stands and colonies (Figs. 3 and 4) and occurring to a maximum depth of 73 m (Fig. 5), indicating this group is a significant component of the mesophotic coral assemblages of the region.

**Table 1:** Staghorn coral species from the shallow reef and mesophotic zones of north-east Australia.

Species	Shallow reefGBR^1^	Shallow reefCoral Sea^1^	MesophoticGBR	MesophoticCoral Sea	Category	Range Size x 10^6^km
*A. abrolhosensis*	-	+	-	R	M	OA
*A. abrotanoides*	+	+	-	-	S	139.71
*A. aculeus*	++	+	+	+	G	119.71
*A. acuminata*	+	+	-	-	S	179.52
*A. anthocercis*	+	+	-	-	S	88.63
*A. aspera*	++	++	-	-	S	66.44
*A. austere*	++	++	R	R	G	151.32
*I. brueggemanni*	+	+	-	-	S	29.55
*A. bushyensis*	R	-	-	-	S	9.49
*A. cardenae*	-	-	+^1^ ^,^ ^2^	-	D	OA
*A. carduus*	+	R	R	R	G	38.56
*A. caroliniana*	+	+	R	R	G	39.95
*A. cerealis*	++	++	+	+	G	129.40
*A. chesterfieldensis*	+	+	+	+	G	10.13
*A. clathrata*	+	+	R	-	M	OA
*I. crateriformis*	R	-	-	-	S	12.11
*I. cuneate*	+	R	-	-	S	30.62
*A. cytherea*	+	+	R	-	M	OA
*A. dendrum*	+	+	-	-	S	9.90
*A. digitifera*	++	++	-	-	S	184.38
*A. divaricata*	++	++	-	-	S	125.02
*A. donei*	+	+	+	+	G	72.04
*A. echinata*	+	+	+	+	G	38.73
*A. elegans*	-	-	+^2^	-	D	OA
*A. elseyi*	++	++	R	-	M	OA
*A. florida*	+	+	R	-	M	OA
*A. gemmifera*	+	+	-	-	S	104.89
*A. glauca*	+	-	-	-	S	88.63
*A. globiceps*	-	R	-	-	S	23.44
*A. grandis*	+	R	-	-	S	51.10
*A. granulosa*	++	++	+	+	G	80.99
*A. horrida*	+	+	R	-	M	OA
*A. humilis*	++	++	-	-	S	157.71
*A. hyacinthus*	++	++	-	-	S	148.12
*A. intermedia*	+	+	-	-	S	100.89
*A. kimbeensis*	-	+	R	+	G	9.90
*A. kirstyae*	+	+	-	-	S	19.18
*A. latistella*	++	++	R	R	M	OA
*A. listeria*	+	+	-	-	S	106.28
*A. lokani*	R	-	-	R	M	OA
*A. longicyathus*	++	++	-	-	S	41.60
*A. loripes*	++	++	+	+	G	121.45
*A. lovelli*	+	-	-	R	M	OA
*A. lutkeni*	+	+	-	-	S	151.27
*A. microclados*	+	+	-	R	M	OA
*A. microphthalma*	+	+	-	-	S	169.32
*A. millepora*	++	++	-	-	S	69.86
*A. monticulosa*	+	+	-	-	S	78.81
*A. multiacuta*	R	-	-	-	S	16.14
*A. muricata*	++	++	-	-	S	181.42
*A. nana*	+	+	-	-	S	56.55
*A. nasuta*	++	++	-	-	S	125.72
*I. palifera*	+	+	-	+	G	81.07
*A. palmerae*	+	-	-	-	S	58.02
*A. paniculata*	+	+	+	+	G	91.03
*A. papillare*	+	-	-	-	S	25.80
*A. pichoni*	-	-	R	-	D	OA
*A. polystoma*	+	+	-	-	S	90.00
*A. pulchra*	+	-	-	-	S	83.21
*A. robusta*	+	+	-	-	S	135.12
*A. samoensis*	+	+	-	-	S	122.40
*A. sarmentosa*	++	++	-	-	S	41.31
*A. secale*	++	++	R	-	M	OA
*A. selago*	+	+	-	R	M	OA
*A. solitaryensis*	+	R	-	R	M	OA
*A. spathulata*	+	R	-	-	S	0.58
*A. speciosa*	+	+	+	+	G	50.87
*A. spicifera*	+	-	-	-	S	77.76
*A. striata*	+	+	-	-	S	111.35
*A. subglabra*	+	R	R	R	M	OA
*A. subulata*	+	+	-	-	S	116.63
*A. tenella*	-	-	+	-	D	OA
*A. tenuis*	++	++	-	-	S	83.66
*A. torihalimeda*	-	-	+^1^	-	D	OA
*A. tortuosa*	+	-	R	-	M	OA
*A. valenciennesi*	+	+	+	-	G	40.05
*A. valida*	++	++	+	-	G	304.07
*A. vaughani*	+	+	R	-	M	OA
*A. verweyi*	+	+	-	-	S	124.65
*A. willisae*	+	-	R	-	M	OA
*A. yongei*	+	-	-	-	S	98.97
**Total**	73	63	31	22		

Specimen-based *Acropora* and *Isopora* species records from the shallow reef (≤ 30m depth) and mesophotic zone (> 30 m depth) zones of the Great Barrier Reef and western Coral Sea. Species recorded exclusively in the shallow reef (S), exclusively mesophotic zone (D), depth generalist (G) and marginal generalist (M), most common (++), rarely recorded (R), omitted from range-size analysis (OA). Global range size and shallow-reef records used WWAC data.

^1^ reported in [17]

^2^ reported in [10]

### Species diversity

Prior to this study staghorn species were reported to be a relatively scarce component of mesophotic diversity [4], with one species documented for the mesophotic zone of Johnson Atoll (Central Pacific) [24], one for the Caribbean [25] and at least three for American Samoa [9]. However these areas are relatively depauperate in shallow water staghorn species, having three, two and 29 species respectively [17]. This study from north-east Australia is the first report of diversity at mesophotic depths from a region with relatively high shallow reef staghorn diversity (76 species [17,18]). Several *Acropora* species are also reported at depths below 30 m [2,10,18,26–29], but these reports do not include estimates of species diversity. We found 38 staghorn species in the mesophotic zone of north-east Australia, including many species with wide Indo-Pacific distributions [17,18], indicating that the group is likely to be a significant component of mesophotic assemblages in other areas of the Indo-Pacific. We found fewer species in the mesophotic zone of the western Coral Sea than in the GBR (22 and 31 species respectively, Table 1), a pattern consistent with results for shallow reef species richness (63 and 73 species respectively, Table 1). The mesophotic zone results are likely underestimates, based upon 27 sites with the sampling effort restricted due to the constraints of sampling at depth. Sampling sites were also restricted in their habitat diversity, with most sites on lower reef slopes in close proximity to emergent reefs due to ROV operational requirements and SCUBA diving logistics. Habitats with a gradual reef-slope, submerged banks and deep sites on the outer barrier remain largely unexplored and have the potential to produce more new mesophotic staghorn records. To illustrate this point, limited sampling of submerged banks in the GBR conducted over 20 years ago described two new *Acropora* species, *A. cardenae* [26] and *A. torihalimeda*, [27] but to date, only a few specimens of each taxon are documented and their geographic distribution is not known [17]. Further data on the biodiversity of corals from deep, inter-reef habitats are required, particularly as these habitats are suggested to be more resistant to global and local stressors relative to shallow water reefs [7,29].

Of the 38 species recorded from the mesophotic zone, 17 were categorized as marginal depth generalists, recorded only on one or two occasions between 30 m and 40 m depth (Category “M”, Tables 1 and 2). Many of the sites sampled were low-latitude (Fig. 1) and on the outer barrier of the GBR or in the Coral Sea, in areas characterized by exceptionally high water clarity [30]. Such conditions are optimal for the penetration of light at depth [31], so that staghorn species normally restricted to the shallow reef by their light requirements may occasionally extend into the upper mesophotic at these locations. Furthermore, very severe tropical cyclones have occurred near some sampling sites within the previous ten years, including Cyclone Yasi which produced severe damage to a site at Myrmidon Reef to depths of 60 m [32]. This system appeared to have produced swells of sufficient energy to move entire sections of reef matrix with corals attached into deeper water and it is possible that despite our precautions, we inadvertently sampled colonies such as these.

**Table 2:** Summary of *Acropora* and *Isopora* species richness for the GBR and Coral Sea.

	Exclusively Shallow reef	Marginal Generalist	Depth Generalist	Exclusively Mesophotic	Total
**GBR**	42	16	16	5 (1)	79 (1)
**Coral Sea**	34	15	16	0 (1)	65 (1)
**Total**	43	17	16	5 (2)	81 (2)

Number of species recorded which were categorized as exclusively shallow reef (≤ 30m depth), exclusively mesophotic zone (> 30 m depth), both zones (depth generalist) and both zones but only rarely to 40 m (marginal generalist), with additional potential new species indicated in brackets.

### New species records

**Fig 6:**
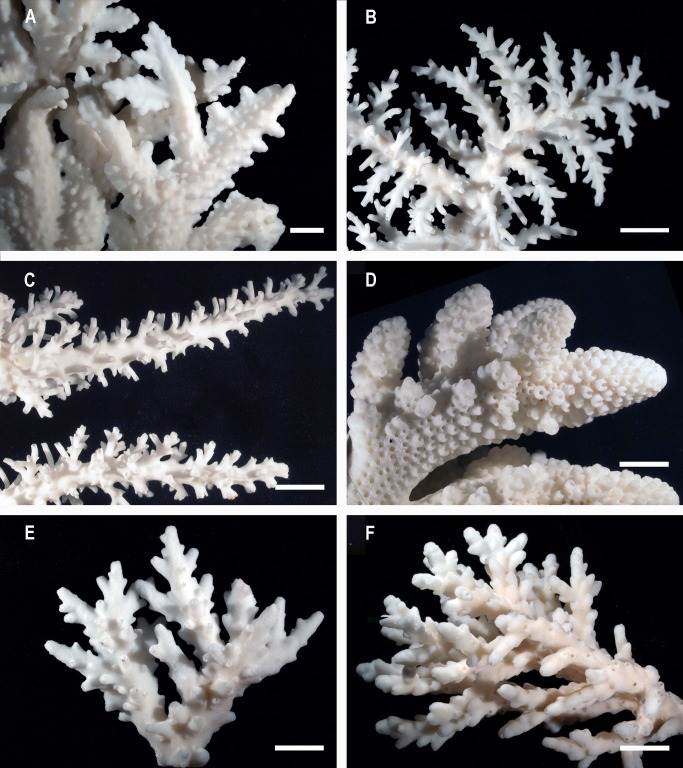
Morphological changes with depth. (A) Deep-water specialists *A. pichoni* collected 40 m and (B) *A. tenella* collected 52 m, (C) depth generalist *A. echinata* collected 40 m (D) typical morphology for inter-tidal species *A. humilis* collected 1 m (E) depth generalist *A. granulosa* from 46 m depth and (F) from 7 m depth. Staghorn corals collected at depths ≥ 40 m generally have laterally flattened branches, a lighter and more fragile skeleton and increased spacing between corallites and branches. Scale bars: 1cm.

**Fig 7:**
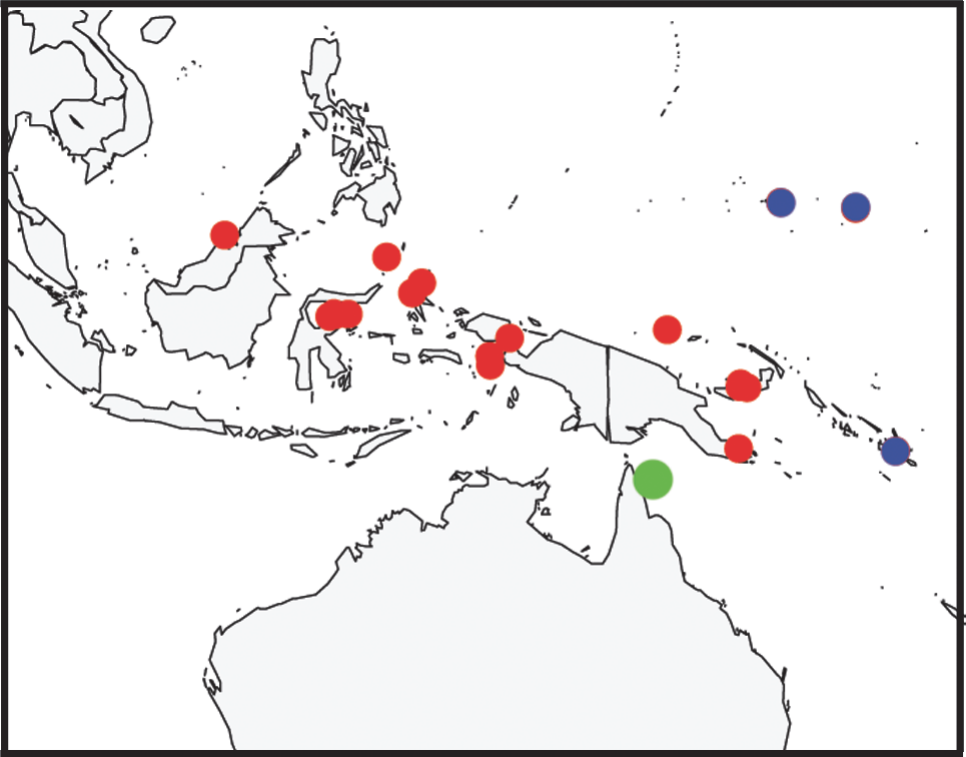
Geographical distribution of *A. pichoni*. Previously recorded for Indonesia (red points, [18]) and considered rare and endangered [29,35], this species has recently been recorded for other areas (blue points [17]) and here for north-east Australia (green point). Three other species found in the mesophotic of north-east Australia have similar increases in their distribution.

Four species were newly recorded for Australia, including *A. elegans*, reported in an initial survey by the authors in [10]. Finding new species records in the GBR and Coral Sea is unusual because reef-building corals, particularly the genus *Acropora*, have been relatively well documented in the region [17, 18, 33, 34], highlighting the dearth of information on coral biodiversity in deeper waters. There is some prior indication that the distribution of one of these species, *A. kimbeensis*, includes north-east Australia (map p.352 [2]), although no data or specimens are provided and *in-situ* identification is difficult for this species [17,18]. The other three species (*A.elegans, A. pichoni, A. tenella*) are distinctive, having unusual morphology (Fig. 6A and B) which is unlikely to have been overlooked and they have probably remained undocumented to date as they were only recorded at or below 40 m depth. Recent data [17,28], along with these new records, extend the geographical range of each species substantially (Fig. 7), as previously they were documented only for Indonesia with one species extending to PNG and another to the South China Sea [18]. In light of these range extensions the classification of these species as rare or globally restricted [29,35] and the IUCN listing as endangered and rare [23] may have to be reassessed. The presence of deep-water “Coral Triangle” species also suggests links between the deep-water fauna of the far northern GBR and Coral Sea and northern Papua New Guinea. Interestingly, the five exclusively deep-water species belong to the *A. elegans* species group (Table 3) and show close phylogenetic affinity [18]. These records increase the number of species of staghorn corals in the region to 81 (Table 1). In addition, we found two potentially undescribed *Acropora* species exclusively below 40 m depth, which will be described pending further analysis. The new records for the region and potential undescribed species restricted to depths greater than 40 m, highlight the need to include the mesophotic zone in assessments of reef species diversity and the management of reef and inter-reef areas.

**Table 3:** Details of mesophotic *Acropora* and *Isopora* species from north-east Australia.

Species	MaximumDepth (m)	ColonyType	SpeciesGroup		Species	MaximumDepth (m)	ColonyType	SpeciesGroup
*A. abrolhosensis*	40	A	*horrida*		*A. lokani*	40	C	*loripes*
***A. aculeus***	60	H/C	*latistella*		***A. loripes***	40	C	*loripes*
***A. austera***	40	A	*austera*		*A. lovelli*	30	A	*lovelli*
A. cardenae*	55	F	*elegans*		*A. microclados*	33	T	*hyacinthus*
***A. carduus***	40	H	*echinata*		***I. palifera***	40	CN	*Isopora*
***A. caroliniana***	40	C	*loripes*		***A. paniculata***	42	P	*hyacinthus*
***A. cerealis***	40	C	*nasuta*		A. pichoni*	40	P	*elegans*
***A. chesterfieldensis***	40	C	*loripes*		*A. secale*	30	C	*nasuta*
*A. clathrata*	40	P	*divaricata*		*A. selago*	40	C	*selago*
*A. cytherea*	38	T/P	*hyacinthus*		*A. solitaryensis*	40	AT	*divaricata*
***A. donei***	45	P	*selago*		***A. speciosa***	60	P	*loripes*
***A. echinata***	60	H	*echinata*		*A. subglabra*	33	H	*echinata*
A. elegans*	55	P	*elegans*		A. tenella*	56	H/P	*elegans*
*A. elseyi*	34	H	*echinata*		A. torihalimeda*	63	F	*elegans*
*A. florida*	40	H	*florida*		*A. tortuosa*	35	A/H	*horrida*
***A. granulosa***	50(73)	P	*loripes*		***A. valenciennesi***	**45**	AT	*muricata*
*A. horrida*	35	H	*horrida*		***A. valida***	40	C	*nasuta*
***A. kimbeensis***	40	C	*nasuta*		*A. vaughani*	40	A	*horrida*
*A. latistella*	40	C	*latistella*		*A. willisae*	40	C	*loripes*

Based on collected specimens (with *cf*. records bracketed), the maximum depth (m), species group [18], and colony type [18] for exclusively mesophotic*, depth generalist and marginal generalist species. Colony type: arborescent (A), plate (P), free living arborescent (F), hispidose (H), arborescent table (AT), table (T), corymbose (C) and cuneiform (CN).

### Mesophotic morphology

*Acropora* colonies from depths 40 m and below generally had laterally flattened branches, a light and fragile skeleton and unusually wide spacing between corallites and branches (Fig. 6). Lateral flattening is reported for other deep-water coral genera and is interpreted as a means of increasing surface area available for interception of light [18,36]. Fine and fragile skeletal structures are also reported for deep-water corals (summarized in [4]) and are proposed as a response to decreased solar irradiance and hydrodynamic energy [37]. Wide spacing between corallites has been reported previously for deep-water staghorn corals [18,38] and low polyp density at depth is reported for other coral genera [4,39]. Staghorn species typical of the shallow intertidal reef have closely packed corallites (Fig. 6D, [18]), which suggests spacing may be related to an environmental factor that varies with depth. Solar irradiation decreases not only with depth but also with latitude, shading and turbidity [40]. However, staghorn corals from shaded, turbid or high latitude shallow reef locations generally do not display unusual corallite spacing (authors’ observations). Water movement, specifically turbulent water motion associated with surface waves, also tends to decrease with depth in many reef areas. Depths below 30 m are unaffected by surface waves in all but the most extreme storm events and current flows are typically low in deep fore reef habitats relative to the shallow reef [41]. As coral polyps do not possess specialized structures for dissolved gas exchange and excretion but rely upon diffusion across the general tissues [42, 43], they may be restricted to a low density in conditions of low water movement. While currents driven by tidal forcing and wind stress can be relatively high in some deep reef habitats [43], periods of slack water in the absence of turbulent wave motion may limit polyp density. In captive environments, shallow reef staghorn corals in particular require a continual high rate of water movement for survival (e.g. [44]). The relation between polyp density and water movement for staghorn corals, and indeed corals in general, requires experimental study.

**Fig 8:**
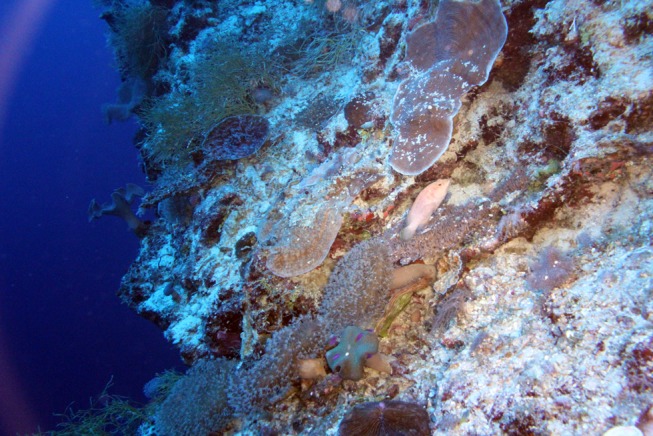
Semi-suspended coarse sediments affecting mesophotic corals. Coarse sands were frequently observed cascading down sloping mesophotic habitats, settling on and partially burying corals. Osprey Reef, Coral Sea at 40 m depth.

In the exclusively mesophotic species, colony shape was restricted to plating and free-living arborescent growth forms (Table 3). The plating morphology found in these taxa was quite different to that found in shallow reef taxa, with plates formed by lateral flattening and spacing of the branches and an increase in corallite spacing (Figs. 3 and 6A, B, E). Depth generalist species displayed hispidose, corymbose, arborescent, plating and rarely arborescent table and cuneiform colony forms (Table 3). The arborescent, digitate and table colony forms, which are common in shallow reef *Acropora* [18], were rare or absent below 30 m depth for the sites surveyed. Interception of light, hydrodynamics, competition and predation likely restrict the colony shape of staghorns [2,18,35,45]. In addition, many lower reef slopes are exposed to coarse, semi-suspended sediments advected down the reef slope. For sites with a steep slope or wall adjacent to shallow reef habitats we frequently found high levels of coarse calcareous sands that cascaded down the slope forming chutes or “rivers of sand” that settled upon corals, partially burying some colonies (Fig. 8). Similar coarse sediments and “chutes” were reported for steep reef slopes at Enewetak Atoll at mesophotic depths [46]. Carbonate productivity of shallow reefs is estimated at up to 110 t ha^-1^ yr^-1^ [47] and much of this material is likely to become coarse sand transported down the reef slope. While horizontal surfaces are needed by mesophotic corals to maximize interception of light at depth [45], such surfaces appear prone to collecting coarse sediments, particularly in habitats below the wave base with low water movement. Accumulation of sediment on coral surfaces is well documented to be deleterious [48], therefore extensive growth of mesophotic corals requires strategies for dealing with down-welling coarse sands. In staghorn corals, the indeterminate and “diffuse plate” morphologies commonly found in the mesophotic *Acropora* species probably minimize sediment accumulation while maintaining adequate photosynthesis. Thin branches and an indeterminate growth form would also allow relatively rapid rates of branch extension and therefore an ability to recover from partial burial. These extreme morphologies allow *Acropora* to extend to depths of 73 m at some sites (Table 3). Reef-building corals extend down to depths of 125 m in this region [5], although the colony morphologies found below 70 m were almost exclusively solid plate and encrusting forms.

### Overlap with shallow reef staghorn fauna

We identify some overlap in shallow reef and mesophotic staghorn faunas, with 21% of shallow reef species also recorded in the mesophotic zone (depth generalists) and a further 22% recorded on only one or two occasions from 30 to 40 m depth (marginal depth generalists, Tables 1 and 2). The overlap in the western Coral Sea was slightly lower, relative to the GBR (12% and 20% depth generalists respectively). Considering the 20 most common staghorn species that comprise a significant component of a wide range of reef habitats in the region, six species or 30% were depth generalists and three marginal generalists (Table 1). For the depth-generalist and marginal generalist species, mesophotic habitats could provide a refuge against shallow-reef disturbances according to the deep reef refuge hypothesis (DRRH, reviewed by [14]). The hypothesis proposes surviving deep water populations would act as a source of larval recruits, aiding recolonization of the shallow reef and preventing localized extinction. Refuge therefore depends upon the ability of depth-generalist and marginal generalist species to successfully reproduce at mesophotic depths. Staghorn corals require sufficient energy for gametogenesis, a series of tidal and lunar cues for synchronising gamete or planulae release [49] and their capacity for self-fertilisation is limited [50]. Therefore, under mesophotic conditions of reduced light [4], reduced lunar illumination, increased hydrostatic pressure and for isolated individuals, the reproduction of some taxa may be impeded. Recruitment to the shallow reef of planulae originating in the mesophotic would also be limited if deep populations represent distinct lineages specifically adapted to low light or different hydrodynamic conditions. The mesophotic individuals of depth-generalist species were morphologically quite distinct (Fig. 6C and D), but the degree to which this is due to phenotypic plasticity rather than adaptive divergence remains unclear. Genetic divergence over depth has been established in both brooding [51–53] and a broadcast spawning coral (i.e. *Montastraea* [54]) and has the potential to result in reproductively isolated populations or limited vertical connectivity in “depth-generalist” corals despite broad bathymetric distributions. Regardless of the potential for mesophotic staghorn populations to aid in rapid shallow reef recovery, the overlap in staghorn coral species diversity between shallow and mesophotic habitats may be important for lineage continuation, safeguarding a reasonable proportion of staghorn coral diversity in deeper water. Of the 22 *Acropora/ Isopora* species groups or lineages identified by [18] from a morphological phylogeny, seven were depth generalists or exclusively mesophotic species (Table 3).

The DRRH suggests that depth generalist species are less susceptible to localised extinction events relative to exclusively shallow reef species (reviewed by [14]). Localised extinction has been documented for *Acropora* in the Marquesas Islands following the last deglacial [55], for *Isopora* in the Caribbean [56] and several coral species in the eastern Pacific following severe ENSO events [57]. Since localized extinction events are a factor constraining geographic range-size [58], depth generalists could be expected to have a larger range-size relative to species restricted to the shallow-reef. However, we found no such signal for staghorn species of the region: depth generalists had a similar mean range-size to exclusively shallow-reef species (86.2 X 10^6^ km^2^ and 85.7 X 10^6^ km^2^ respectively, t-test = -0.02, p = 0.98).

The small proportion of depth generalist species and an analysis of range-size suggests that the potential for mesophotic populations to aid in rapid shallow reef recovery may be limited for staghorn corals in the region. However, this study, is based upon just 27 mesophotic zone sites and the range-size analysis does not account for the many other factors determining species range (e.g. [58]), so clearly this important hypothesis requires further testing. Although the proportion of depth-generalist species is relatively low, deep reefs may nonetheless provide protection for several staghorn coral lineages.

### Conclusions

Despite the limitations of reduced light and the influence of down-welling sediments, the mesophotic zone provides many advantages for reef-corals including reduction in extremes of temperature, solar radiation and hydrodynamic energy and low levels of disturbance from bleaching, storm damage and many human impacts. We found surprisingly high diversity of staghorn corals at mesophotic depths of the Great Barrier Reef and the western Coral Sea, demonstrating that these corals represent a significant component of upper mesophotic communities (30–60 m). The relatively high abundance of staghorn corals at some locations attest to the favourable conditions that can occur at mesophotic depths (Fig. 2), whereas the extremely large size of many colonies (Figs. 3 and 4) are indicative of low levels of disturbance. Staghorn corals clearly are a significant component of many mesophotic coral assemblages on the GBR and Coral Sea. Previous assumptions of low diversity of this important group in deep habitats highlights the paucity of information on deep-water coral reefs, even in well-studied regions such as the GBR.

## Materials and Methods

Observations and sample collections were made during six expeditions to the GBR and atolls of the Coral Sea Commonwealth Marine Reserve from 2010 to 2013 (Fig. 1), with the majority of data obtained during the “Catlin Seaview Survey” expeditions in 2012. An initial survey was conducted in 2007 and is reported in [10]. Twenty seven sites were studied and were usually selected based on (and therefore biased towards) a steep bathymetric gradient to provide access to mesophotic depths while still allowing shallow-water anchoring (required for ROV operations). Sites were surveyed down to 40 m using SCUBA, and from 40 to 150 m depth using a Seabotix vLBV300 or LBV200 with manipulator. Accurate in-situ identification is often not possible for many staghorn species [18] and was particularly difficult in this study due to the unusual morphology of many mesophotic colonies, as well as constraints associated with SCUBA (i.e. bottom time) and ROVs (i.e. video quality). Therefore, for each record we removed a small sample, recorded depth of occurrence and where possible, photographed the colony and corallites *in situ*. To minimize the chance of sampling corals that had been transported down the reef slope from shallower depths, only colonies attached to the substrate in a normal orientation that appeared healthy and free of bleaching, fouling and mechanical damage were recorded. On return to the surface, specimens were cleaned in household bleach solution (4% hypochlorite) for 36–72 h, rinsed in freshwater and dried. Where possible, samples of species found in the mesophotic zone were also collected in the adjacent shallow-reef to enable comparison of mesophotic zone and shallow-reef morphology.

In the laboratory, samples were identified according to [17,18] using a Wild M5 binocular microscope and by comparison with specimens from the World-Wide *Acropora* Collection (WWAC), housed at the Museum of Tropical Queensland, Queensland Museum. This collection consists of approximately 23,000 specimens collected at 1,800 sites across the majority of coral reef areas of the world [17,18]. The WWAC database was also used to determine shallow-reef species records, most common species and for additional mesophotic records from the region. The 20 most common shallow-reef species were estimated from the total number of specimens of each species in the WWAC that were collected at a depth of 30 m or less in north-east Australia. To determine changes in morphology with depth we also examined specimens from the WWAC from shallow reef localities in close proximity to our sampling sites. Characters and character states used for this comparison are described in [17,18]. Samples collected during the study are lodged in the WWAC under accession numbers: G63588-63666, G63786, G63787, G63801-63826, G64589, G64774-G64802, G65114-65173, G65371-65422, G65493-65627, G65744, G66040-66054, G66966, G67398, G67403 and G68542—G68735.

Statistical analyses were conducted using the Fossil package [59] implemented in R v. 3.0.2. Sampling effort varied between sites due to difficulties in working at depths below 30m so that estimating total species richness and differences between sites was not possible. Species range-size (Table 1) was estimated using distribution data from the WWAC. For each species, range was approximated as two minimum convex hulls, one for the Pacific Ocean basin and the other the Indian Ocean basin, and the area of these polygons on the earth’s surface estimated according to [59]. The boundary between ocean basins was defined as a line passing north through Thailand, Malaysia and Sumatra, to West Java, eastwards to E. Timor and south crossing the Australian coast around the West Australian border, intersecting the positions: 98.9°E, 40°N; 98.9°E, 9.0°N; 129°E, 7°S; 129°E, 40°S. The mean range-size of 16 depth-generalist species was compared to that of 44 species restricted to the shallow-reef (Table 2) using a student’s t test. Species categorized as marginal depth generalists (Table 1) were omitted from the analysis. Fig. 1 was created using ArcGIS.

### Ethics Statement

Permits for collecting and research were obtained from the Great Barrier Reef Marine Park Authority (G11/34722.1, G10/33363.1, G10/33786.1, G12/35281.1) and Department of Environment (018-RRRW-100917-01, AU-COM2010085, 018-CZRS-1207626-01, 018-RRRW-131031-01, AU-COM2012-151 and AU-COM2013-226).
